# Ductile Fracture Behavior of Notched Aluminum Alloy Specimens under Complex Non-Proportional Load

**DOI:** 10.3390/ma12101598

**Published:** 2019-05-15

**Authors:** Łukasz Derpeński

**Affiliations:** Faculty of Mechanical Engineering, Bialystok University of Technology, 15-351 Bialystok, Wiejska 45C, Poland; l.derpenski@pb.edu.pl

**Keywords:** ductile fracture, complex load, non-proportional load, specimen with notches, biaxial loading, aluminum alloy

## Abstract

The paper presents an experimental investigation of the ductile fracture of specimens with different circumferential notches. Specimens made from ENAW_2024-T351 aluminum alloy were subjected to non-proportional tension–torsion loading. The tests were carried out on an MTS testing machine coupled with the ARAMIS 3D 4M vision measuring system, enabling simultaneous non-contact tracking of the elongation and torsional angle of the measurement base. Depending on the assumed notch radius and the non-proportionate load scheme, the critical tensile force and torsional moments that caused the fracture initiation of the specimen were determined. A significant effect of load configurations and notch radius on the shape of the fracture surface as well as the fracture mechanisms causing the failure of specimens was demonstrated. The equation describing the configuration of critical loads for specimens with different notch radii was applied.

## 1. Introduction

Structural elements should be characterized by proper strength and reliability in their working life. A fracture tolerant design and their inspection (e.g., non-destructive) are key tasks in ensuring that structures operate safely for extended periods of service [1,2,3]. In particular, this applies to elements with notches causing stress and strain concentration. In recent years, many authors have executed a number of experimental studies aiming to characterize the plastic behavior of metals under complex non-proportionate loads. In previous papers [4,5,6,7,8,9,10], it has been underlined that the stress triaxiality and the Lode parameter are the two main factors that influence the ductility of metals. In reality, monotonic load tests for different geometries under different load conditions (uniaxial tension, pure shear, proportional, and non-proportionate tension with torsion, uniaxial compression) produce different behaviors in the same material [11,12,13,14]. Nevertheless, the literature still lacks a proper characterization of damage evolution and fracture for non-proportional load paths. In recent years, many researchers [15,16,17,18] have conducted a series of experiments to determine the impact of the load path on ductility. These tests involved the use of several non-proportional load paths as a result of the combinations of tension and torsion load and of compression and torsion load on cylindrical specimens made of steel and aluminum alloy. The aspect that has emerged from previous work is that the fracture deformation is greater when the load is proportional, suggesting that the damage accelerates when non-proportional loading conditions are triggered. In the paper [10], non-proportional tests were carried out using different tensile and torsion combinations on dedicated specimens. The authors came to the conclusion that a linear law was not adequate to take into account the effects of non-proportional loading. A series of experimental studies determining the influence of different states of stress and load paths on the process of ductile fracture is presented in [19,20]. Specially developed specimens (X0-specimen) characterized by four independent notches generating the stress level were used for this purpose. The proposed type of specimens allowed the simultaneous examination of a wide range of stress states and could be used for different load paths. For different proportional and corresponding non-proportional loads, the authors presented the effect of the load path on the process of ductile fracture. Haltom et al. [21] presented studies aiming to establish the degree to which the material could be deformed under complex shear and tensile loads. The tests were carried out for tubular specimens made of 6061-T6 aluminum. The experimental results showed that the failure strain monotonically increased with the decrease of the mean stresses, which differed from the previously presented results in [22] for aluminum alloys. In addition, the measured failure strains were significantly greater than those measured earlier. Investigating the effect of load variability, Roth and Mohr [23] presented the effect of the strain rate on ductile fracture initiation in advanced high strength steel sheets performed on flat specimens with a central hole and different notches. The experimental results showed that the ductility increased by as much as 60% when the loading speed increased from 7.6 × 10^−6^ m/s to 4.2 m/s. Toribo et al. [24] analyzed the influence of loading rate on the fracture process of bluntly notched specimens of pearlitic steel under a hydrogen environment. Results indicate that the location of the fracture zone directly depends on the loading rate. For slow rates, such a zone is placed in the axis of the specimen. On the other hand, in the case of high testing rates, the fracture initiates in the vicinity of the notch tip. They suggested that notch machining could affect the generation of dislocations only in the material at the vicinity of the notch tip as the main dislocations presented in the material were due to cold drawing. According to [25], the local stress in the notch vicinity is not affected by screw dislocations, but the position of the edge dislocations influences the stress state distribution. They also pointed out that the key issue in the studies with notched geometries is the triaxial stress state generated in the material when an external loading is applied [26], which directly affects the distribution of stress within the material, and fracture behavior. Person et al. [27] evaluated the applicability of the strain energy density (SED) approach for the assessment of the fracture strength of experimentally tested elements made of polyetheretherketone (PEEK) subject to corrosion. Various notch geometries under different strain rates and environmental conditions were simulated and showed good agreement with the experimental results. They estimated that the fracture behavior for specimens with different notch radii could be predicted with a discrepancy lower than ±10%. Shokuhfar and Nejadseśli [28] compared the effect of high plastic deformation and heat treatment on the tensile and fracture properties of 6061 aluminum alloy. It was observed that the yield point and ultimate strength significantly increased after two passes in the extrusion process. A series of uniaxial, biaxial, and triaxial tests were carried out in [29] to investigate the effects of the stress state on the fracture of metallic materials, particularly when the plasticity was highly constrained. Khan et al. [30] presented new experiments including pure torsion and uniaxial tension, followed by the torsion and non-proportional biaxial compression of ENAW_2024-T351 aluminum alloy. The aim of this research was to establish a universal, accurate, and efficient fracture criterion for ductile metals. The influence of the load course on ductile failure has been studied in detail for the example of sheet forming [31,32,33] where it was found that the failure strain generated at the necking was strongly dependent on the load path. It should be noted that the existing experimental research is still being developed and new experiments are being created to discover the phenomena associated with the ductile fracture of materials.

This paper presents the results of experimental tests of fracture behavior for various specimens with circumferential notches under monotonic complex non-proportional loads. Studies have shown the significant influence of loading path and notch radius on the shape of the fracture surface. For 10 load configurations from pure torsion to pure tension, it was observed that the shape of the fracture surface changed from planar to a cup-cone shape. This was only obtained when the tensile load was primary. When the notch radius increased, the shape of the fracture surface became more irregular. The critical values of the tensile forces and torsional moments causing fracture initiation for various specimens and load configurations were determined and are described by a simple equation. The presented results also pointed out that complex non-proportional loading would activate the fracture initiation process faster than the complex proportional loads.

## 2. Material Properties

**Figure 1:**
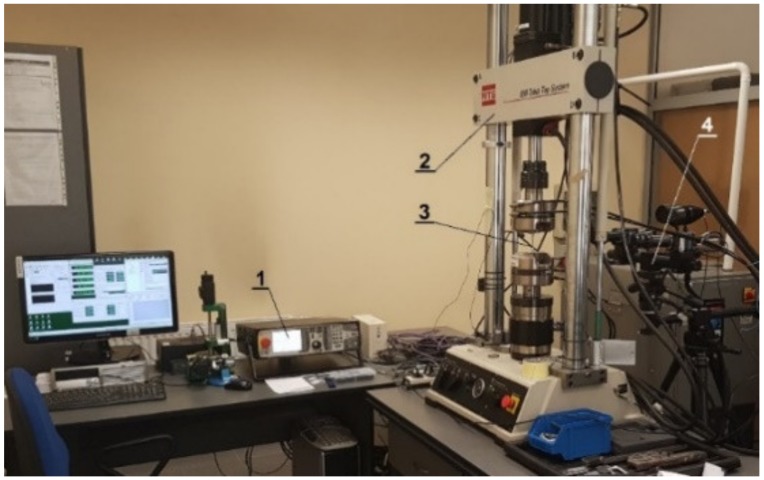
Station for the experimental tests (1—FlexText SE40 controller; 2—testing machine; 3—specimen; 4—ARAMIS 3D 4M system).

The investigations were performed on an ENAW_2024-T351 aluminum alloy supplied as 20-mm-diameter cold-drawn rods. The chemical composition is presented in Table 1. Experimental tests were carried out on an MTS 809.10 two-axis testing machine (Eden Prairie, MN, USA) controlled by the FlexTest SE40 system (Figure 1).

**Figure 2:**
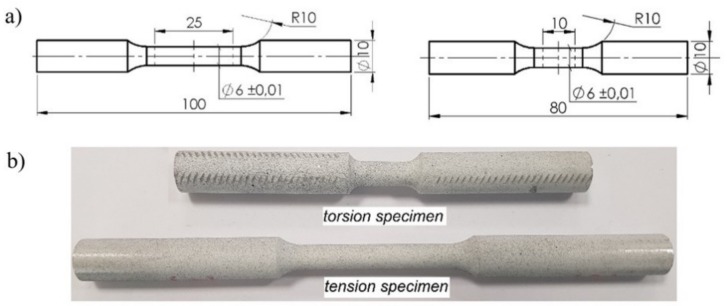
Smooth specimens of the ENAW_2024-T351 aluminum alloy used in tests. (**a**) Dimensions (unit: mm), (**b**) specimens.

The basic strength parameters and the relationships between the tensile force and the elongation of the gauge length of specimen *F*(*u*) and between the torsional moment and the torsional angle of the measuring base *M*_s_ (*φ*) were determined using the axisymmetric specimens presented in Figure 2. In the investigations, cylindrical specimens with an original diameter of *ϕ* = 6 mm were used and the original gauge length was *L*_0_ = 25 mm in the case of a tensile specimen and *L*_0_ = 10 mm in the case of a torsion specimen. The smaller gauge length of the torsional specimen was due to the limited torsional range of the testing machine and the ARAMIS measuring system. For a specimen gauge length equal to 25 mm, the maximum torsional angle was approximate to 140°. The maximal torsional angle for the measuring system was equal to 110°.

**Figure 3:**
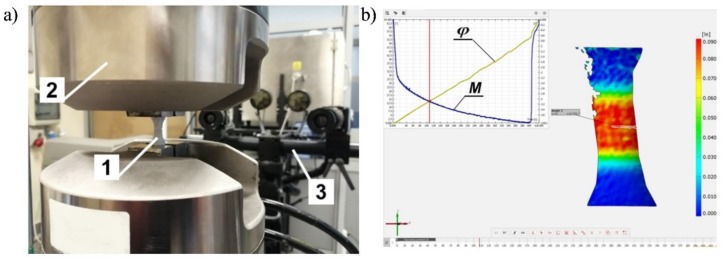
(**a**) The specimen in the machine’s grip with a sprayed external surface during the torsional test (1—specimen; 2—hydraulic grip; 3—ARAMIS 3D 4M system), (**b**) Vision system dialogue (ARAMIS 3D 4M).

All specimens were made from the same rod. The tests were carried out for a given linear velocity equal to 0.1 mm/s and an angular velocity equal to 0.2 rad/s. The tests were carried out until the specimens failed. The obtained data were required to determine the relationship between the stresses and strains, σ(ε) and τ(γ), throughout the entire load range up to the failure of the specimens. The ARAMIS 3D 4M vision system for non-contact three-dimensional deformation measurements was used to continuously monitor the shape change of the specimen’s gauge length during the tests. Therefore, each test specimen was suitably prepared by spraying powder on its external surface to allow for the correct reading of the device (Figure 3). Tests were carried out three times for each load.

**Figure 4:**
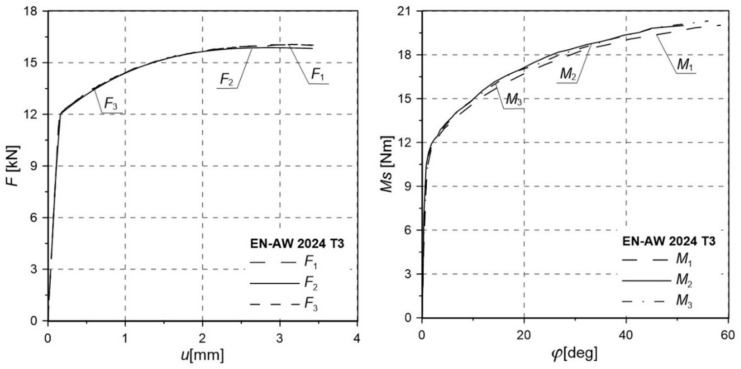
Relationships between tensile force and elongation *F*(*u*) and torsional moment and torsional angle *M*(*φ*) of the measuring base for smooth specimens.

The relationships between tensile force and elongation *F*(*u*) and between the torsional moment and torsional angle *M*_s_(*φ*) of the specimen were obtained (Figure 4). Values of the critical tensile force *F*_c_, the maximum elongation *u*_c_, the critical torsional moment *M*_c_, the critical torsion angle *φ*_c_, and the measurable diameter after the test *d*_f_ are shown in Table 2. In addition, the critical failure strain was calculated according to the relationship proposed in [34]:

(1) $$\varepsilon_{\text{f}}=2\ln\frac{d_{0}}{d_{\text{f}}}$$

It should be noted that the shapes of the curves *F*(*u*) and *M*(*φ*) are both characterized by a mild course of hardening across almost the whole range. In the case of the *F*(*u*) plot, a slight decrease in the value of force was observed at the moment of failure in relation to the maximum value. The presented results show that the loading force increased across almost the whole range, showing only a slight decrease in the final phase of the loading process. The decrease in load was also the result of a reduction in the area obtained in the gauge length of the specimen.

**Figure 5:**
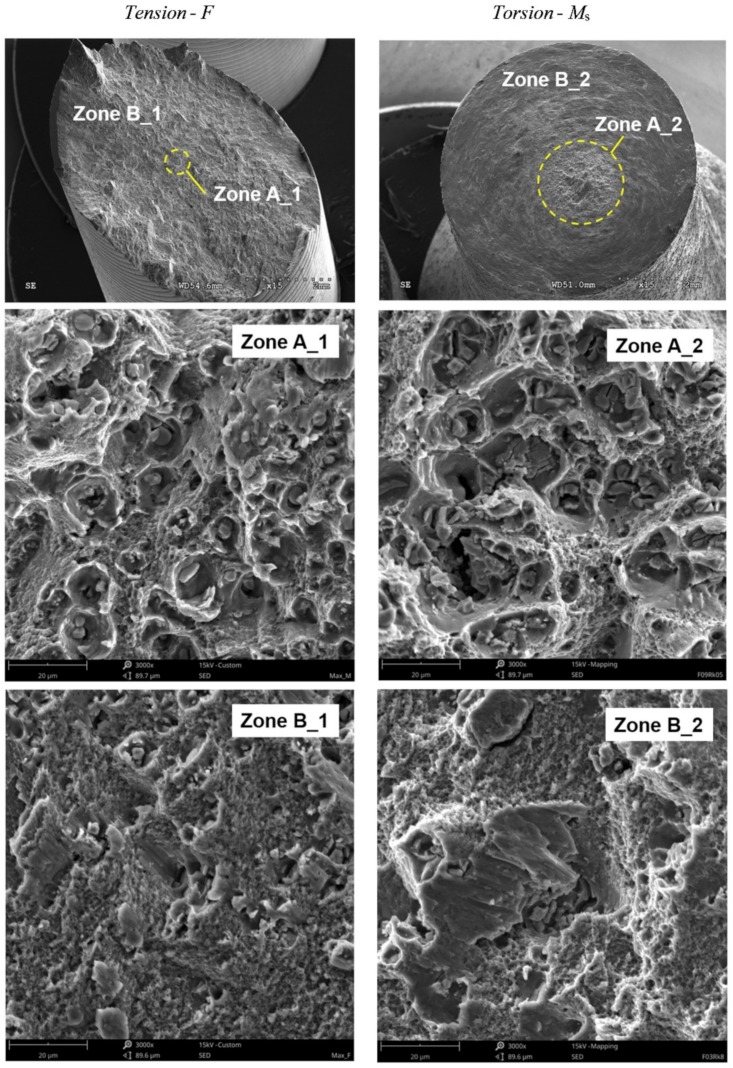
Fracture surface of the smooth specimens.

Figure 5 shows the fracture surfaces of the specimens obtained from the tensile and torsional tests.

From the obtained fracture surfaces of the specimens (Figure 5), a working fracture mechanism could be determined in dependence on the working load. In the case of a tensile test, it should be noted that the fracture surface was almost completely covered with a plane sloping at an angle to the plane perpendicular to the axis of the specimen. Only a small area lying on a plane perpendicular to the axis of the specimen, located in its central part, could be extracted. In this zone (Figure 5, Zone A_1 and Zone A_2), dimples were visible after the deformation of pores in the load direction, which may indicate the dominance of the maximum principal stresses (or strains) in the fracture process. In the remaining part of the fracture surface sloping at an angle, the obtained dimples showed that the maximum shear stresses determined the failure (Figure 5, Zone B_1 and Zone B_2). In the case of a fracture surface obtained from the pure torsion test, only one surface was visible, which was perpendicular to the axis of the specimen. On the prevailing part (Zone B_1, Zone B_2), visible dimples sloped at an angle and had a slender and flattened shape. This was the result of the working load. On this surface, we could also distinguish a small area located in the central part of the fracture surface where we could see a clear difference in the shape of the dimples formed in the final process of specimen decohesion. Their shape was similar to the dimples located in Zone A, which indicates the effect of another fracture mechanism related to the existence of a macro-crack at the circumference of the specimen.

## 3. Investigation of Ductile Fracture of Notched Specimens under Complex Load

The machine used for testing allowed simultaneous and independent control of the tensile and torsional loads in any configuration in the entire load cycle. Due to the large range of torsional angle (by up to 65°–75°) and the elongation of the measuring base up to 1.5 mm, the ARAMIS 3D 4M vision system for non-contact three-dimensional deformation measurements was used in the research process. 

The essential stage of experimental research was to determine the effect of complex non-proportional tensile-torsional loads on the fracture process of axisymmetric specimens with circumferential notches. In this research, 10 configurations of the loads were assumed (Table 3). Load control was adopted in the form of force and torsional moment (*F*, *M*_s_). The load cycle consisted of two load segments generated directly one after other. After reaching the critical value, the primary load stayed under a constant level and at the same moment, the second type of load was activated and realized until specimen failure.

**Figure 6:**
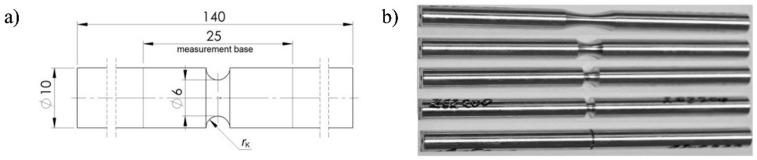
(**a**) Dimensions of specimens used for testing (unit: mm), (**b**) real specimens used in tests.

In the investigations, notched specimens with radii *r*_K_ equal to 0.5, 2, 4, 8, and 30 mm and with a diameter in the notch root of *ϕ*_K_ = 6 mm (Figure 6) were used. The radii of notches were chosen to obtain different distributions of stress and strain in the specimens. The starting material was the same *ϕ* 20-mm drawn bar made of the aluminum alloy ENAW_2024-T351, for which basic strength parameters were determined. The specimens were obtained as a result of machining using CNC machine tools. The shape of the notches was checked by optical microscopy. The manufacturing error was within ±0.01 mm for the notches and ±0.02 mm for the diameter in the notch root.

**Figure 7:**
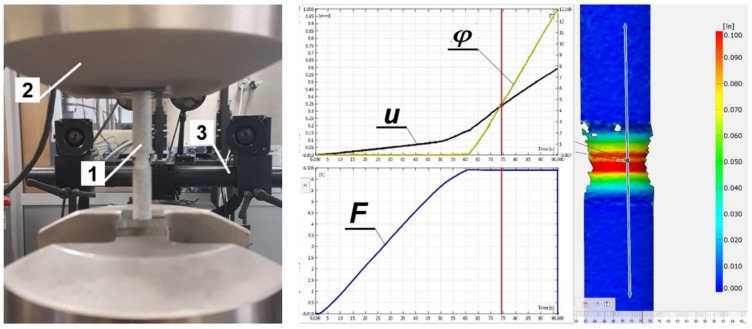
Specimen with notch of radius *r*_K_ = 8 mm with markers (1—Specimen, 2—Hydraulic grip, 3—DIC ARAMIS 3D 4M), vision system dialogue DIC ARAMIS 3D 4M.

Specimens were fastened in hydraulic grips equipped with knurled jaws adapted for fixing axisymmetric elements (Figure 7). In order to exclude displacement and rotation of the specimen in the grips, pre-clamping of nearly 25.0 MPa was used. The shape of the knurls used and the clamping force eliminated the displacement of the specimen relative to the grips during the test.

In the research, it was assumed that the point separating the load segments was the critical value of the tension force $F_{C}^{i}$ or torsional moment $M_{C}^{i}$ as a result of the adopted load configuration. The elongation and torsional angle of the measuring base were measured using the ARAMIS 3D 4M vision system. The force was read from the dynamometer and the moment was read from the torsional sensor located at the head of the testing machine. The tests were carried out until the failure of each specimen, and all notches were entirely in the area of the measurement base. It was assumed that the fracture initiation moment was characterized by a visible drop in the value of the force or torsional moment. The fracture of specimens always occurred in the area of this base. Tests were carried out three times for each specimen. The table below presents the scheme for calculating the critical values of loads that changed the type of load. Therefore, in the case of $P_{n}^{1}$, the specimen was loaded under tension until failure; in the case of $P_{n}^{3}$, the specimen was loaded under tension to half the value of the force obtained during uniaxial tension, and in the next sequence, was loaded under torsion to the failure. Steps similar to the above were followed in the case of the assumed value of torsional moment. In the case of $P_{n}^{6}$, the specimen was loaded under torsion to failure, and in the case of $P_{n}^{8}$, the specimen was loaded under torsion to half the value of the maximum moment obtained during uniaxial torsion, and then the specimen was loaded under tension to failure.

For each of the notches in the entire load range, the tensile forces, torsional moments, elongation, and torsional angle of the measuring base were registered.

**Figure 8:**
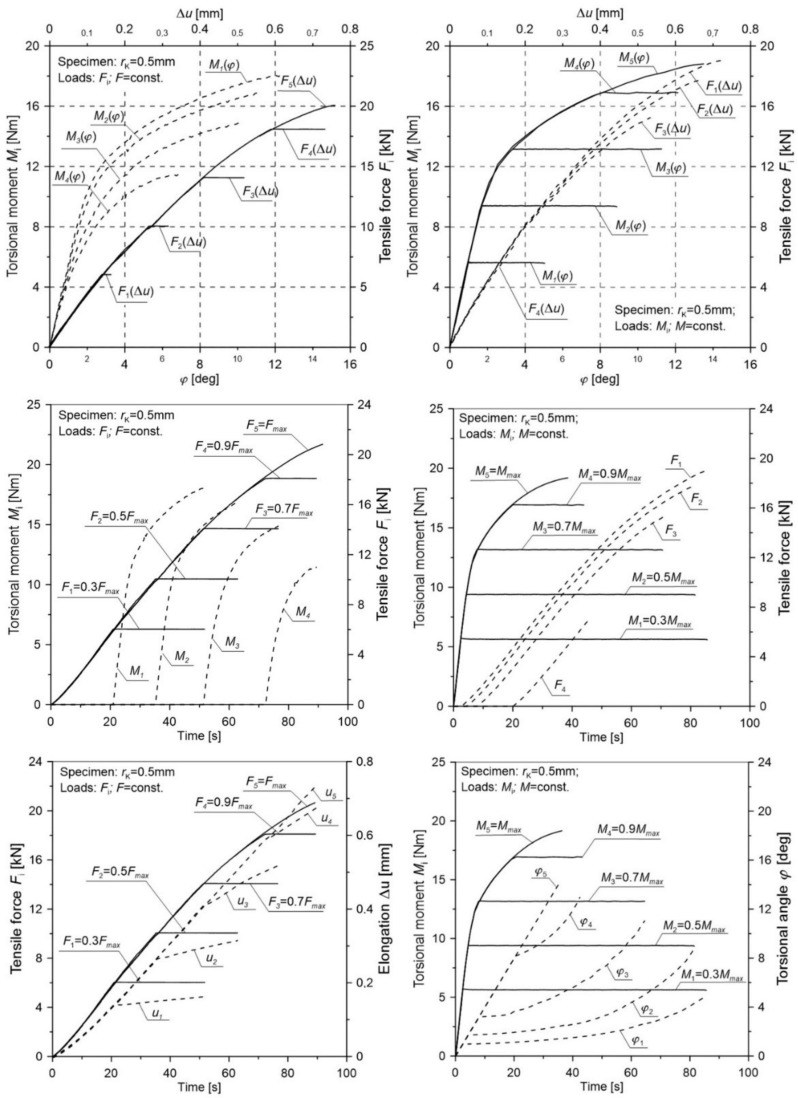
The distribution of the tensile force, torsional moment, elongation, and torsional angle of the gauge length of a notched specimen with a radius *r*_K_ = 0.5 mm for various load configurations.

**Figure 9:**
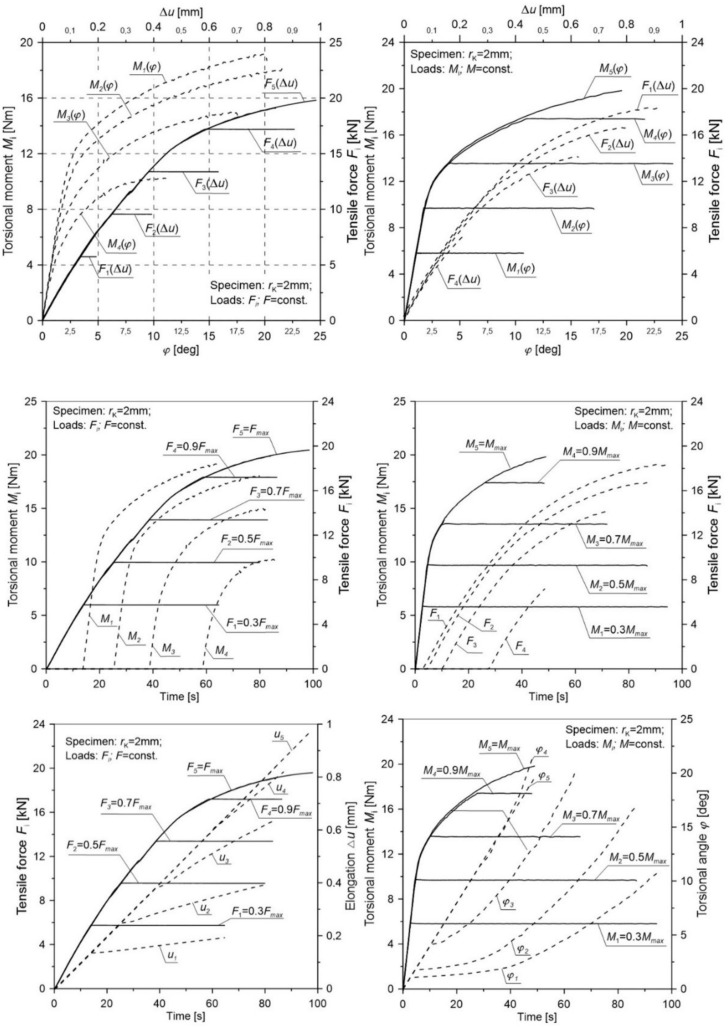
The distribution of the tensile force, torsional moment, elongation, and torsional angle of the gauge length of a notched specimen with a radius *r*_K_ = 2 mm for various load configurations.

**Figure 10:**
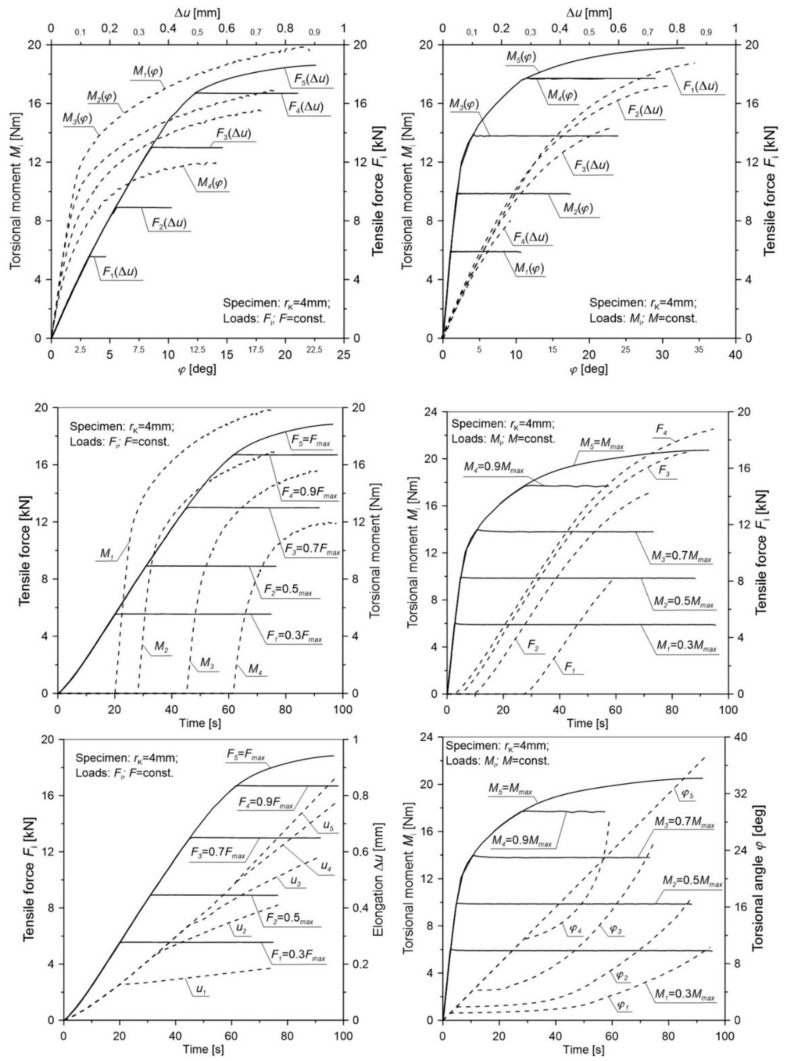
The distribution of the tensile force, torsional moment, elongation, and torsional angle of the gauge length of a notched specimen with a radius *r*_K_ = 4 mm for various load configurations.

**Figure 11:**
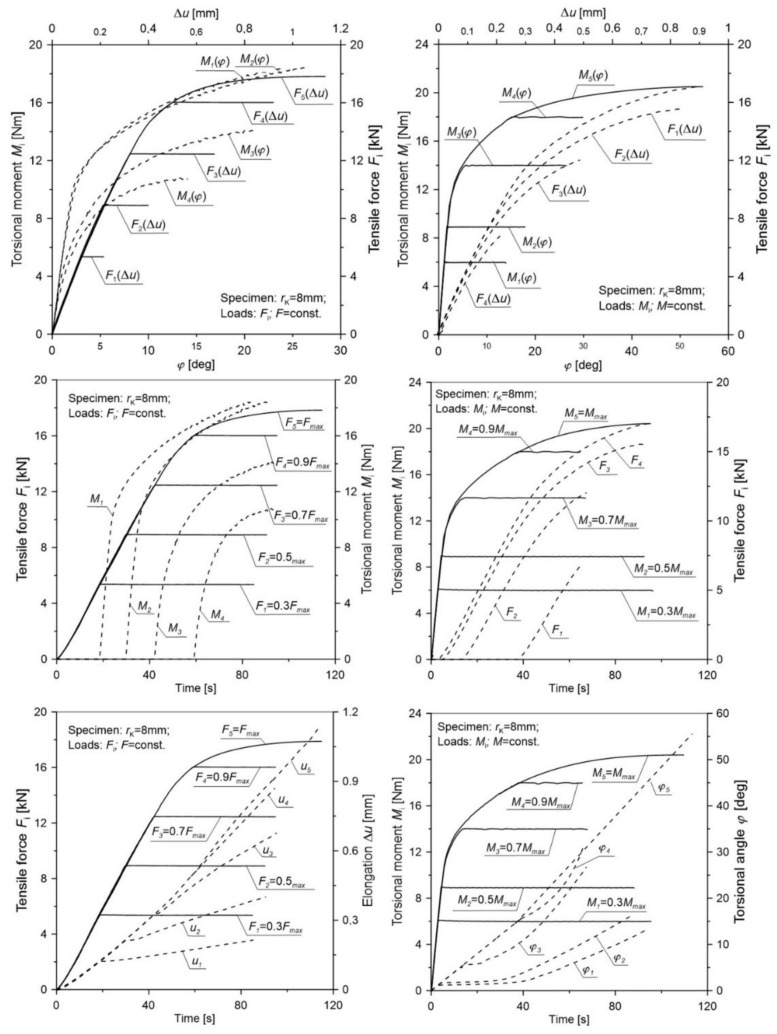
The distribution of the tensile force, torsional moment, elongation, and torsional angle of the gauge length of a notched specimen with a radius *r*_K_ = 8 mm for various load configurations.

**Figure 12:**
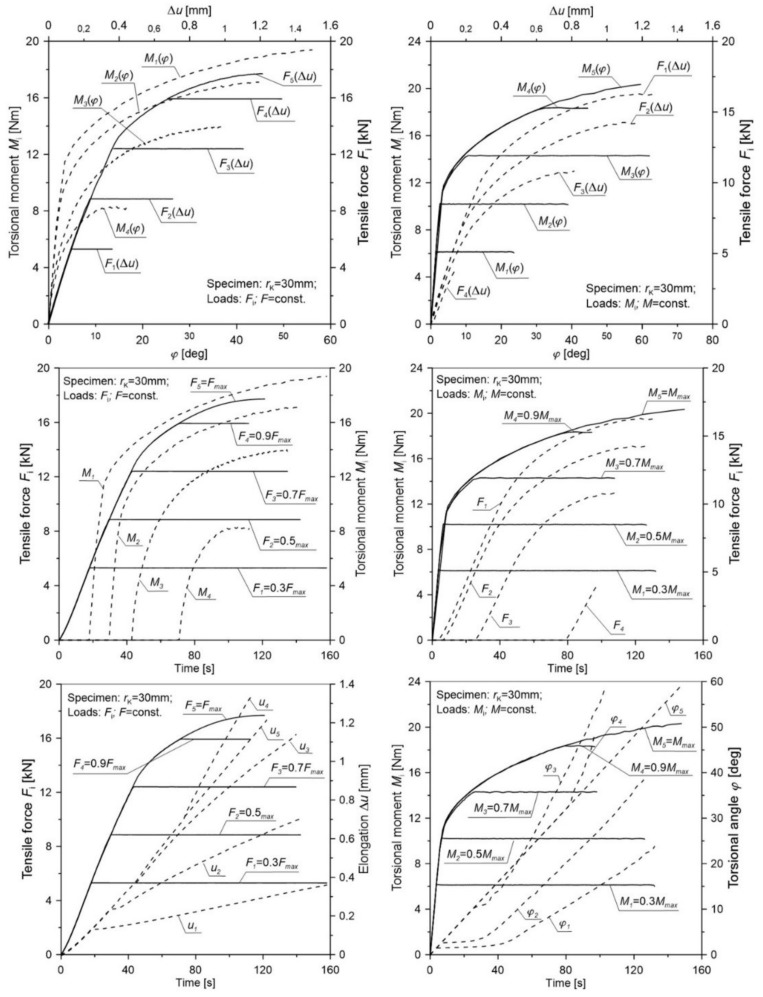
The distribution of the tensile force, torsional moment, elongation, and torsional angle of the gauge length of a notched specimen with a radius *r*_K_ = 30 mm for various load configurations.

Table 4 presents the averaged values for the elongation ${\bar{u}}_{c}$ and torsional angle ${\bar{\varphi }}_{c}$ of the gauge length and tensile force ${\bar{F}}_{c}$ as well as the torsion moment ${\bar{M}}_{c}$ for notched specimens in various load configurations. In order to transparently reflect the selected load scheme additionally in the figures (Figure 8, Figure 9, Figure 10, Figure 11 and Figure 12), the load (tensile force *F* and torsion moment *M*) as well as the elongation *u* and torsional angle *φ* with respect to the duration of the test were presented.

The obtained results (Figure 8, Figure 9, Figure 10, Figure 11 and Figure 12) for non-proportional tension and torsion loads describe the interaction between each other up to failure of the specimen. It was noticed that in the case when the primary load was the tensile force (configurations $P_{n}^{2}$ to $P_{n}^{5}$) for all load configurations and all notch radii, the secondary load course (*M*_s_) showed a nonlinear character. The start of its working depended on the level of the primary load. In the case of the opposite situation when the initial load was the torsional moment (cases $P_{n}^{7}$ to $P_{n}^{10}$), among all considered configurations, the secondary load (force *F*) showed a linear character until failure only for the case $P_{n}^{10}$. This indicates that with such a high primary load level ($M_{C}^{6}=0.9M_{\max}$), only a small part of the tensile force is needed to start the fracture process. For all tested notches, it was also observed that for configurations $P_{n}^{7}$ to $P_{n}^{10}$ in the moment when the load changed from the primary one (*M*_s_) to the secondary one (*F*), a nonlinear increase in the torsional angle (*φ*) occurred until failure. It should be noted that an increase of the angle occurred at a constant level of torsional moment. This behavior may be the result of an easier dislocation mobility on the slip planes caused by the effect of the increasing secondary load at a constant primary load. The same situation was observed in cases $P_{n}^{2}$ to $P_{n}^{5}$, where at a constant level of force *F* load, the elongation of the measuring base *u* in the tested specimen increased linearly.

On the basis of the results presented in Table 4, it was also observed that the increase in the notch radius decreased the critical tensile force (for load case $P_{n}^{1}$) around 15%, and the critical torsional moment (for $P_{n}^{6}$) increased by approximately 9%. In the case of elongation u, they increased up to 50% (for $P_{n}^{1}$), and the torsional angle of the specimen for the load case (for load case $P_{n}^{6}$) increased to around 65%.

**Figure 13:**
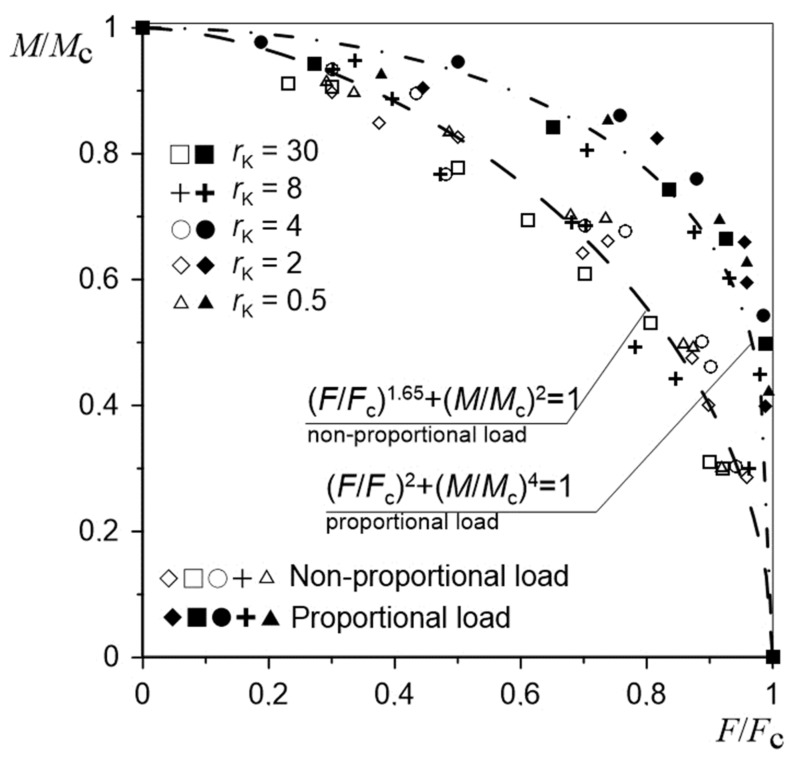
Tension force–torsion moment graph for specimens with notches for various load cases.

Figure 13 presents the values of tensile forces $F_{i}^{}$ and torsional moments $M_{i}$ causing fracture initiation in notched specimens with different radii for different load configurations. These were compared with critical values of proportional loads [12] and can be described in an equally simple way by the following equation:

(2) $${\left(\frac{F_{i}}{F_{c}}\right)}^{A}+{\left(\frac{M_{i}}{M_{c}}\right)}^{B}=1$$

where *F*_c_ is the critical force for uniaxial tension (case $P_{n}^{1}$) and *M*_c_ is the critical torsion moment for uniaxial torsion (case $P_{n}^{6}$). On the basis of the research carried out and presented in [12], the exponential parameters (Table 5) assumed the following values.

Based on the obtained results and comparing them with the results for complex proportional loads (Figure 13), it was observed that the non-proportional load scheme was less advantageous in transferring the applied loads. The tested load behavior scheme was the worst case of the complex loads. The line describing the distribution of critical *F* and *M* values in the non-proportional load configuration presented in the diagram was located below the line designated for proportional loads. This suggests that the complex state of non-proportional loads will activate the fracture initiation process faster than the complex state of proportional loads. In the case of the tested specimens and scheme of loads, it was noticed that for configurations corresponding to half of the critical loads, the transfer of load was nearly 20% lower for complex non-proportional loads than for proportional loads. The obtained results will enable the estimation of the indirect configurations of loads (from non-proportional to proportional) causing the fracture initiation of elements with notches made of the ENAW_2024-T351 aluminum alloy.

**Figure 14:**
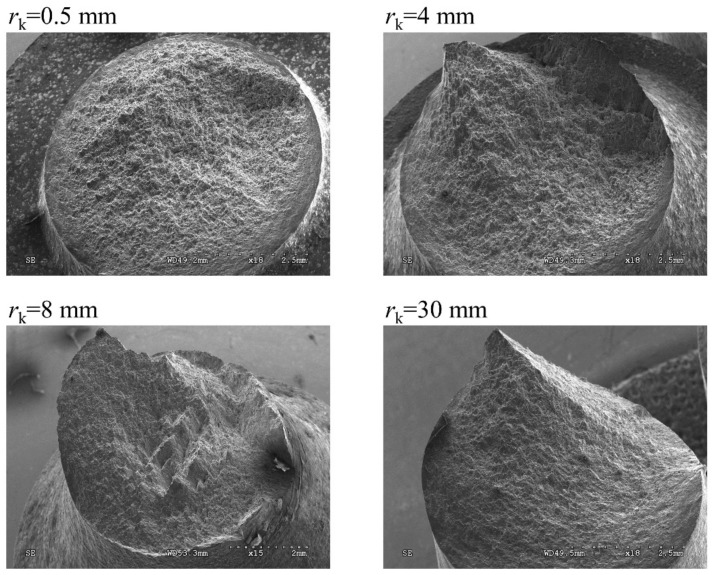
Fracture surface for the load configurations of $P_{n}^{3}$ for different notched specimens.

Figure 14 shows the effect of various notch radii on the fracture surface shape for load configuration $P_{n}^{3}$. It was observed that the fracture surface became more irregular when the notch radius increased and was characterized by a multi-surface of shape.

**Figure 15:**
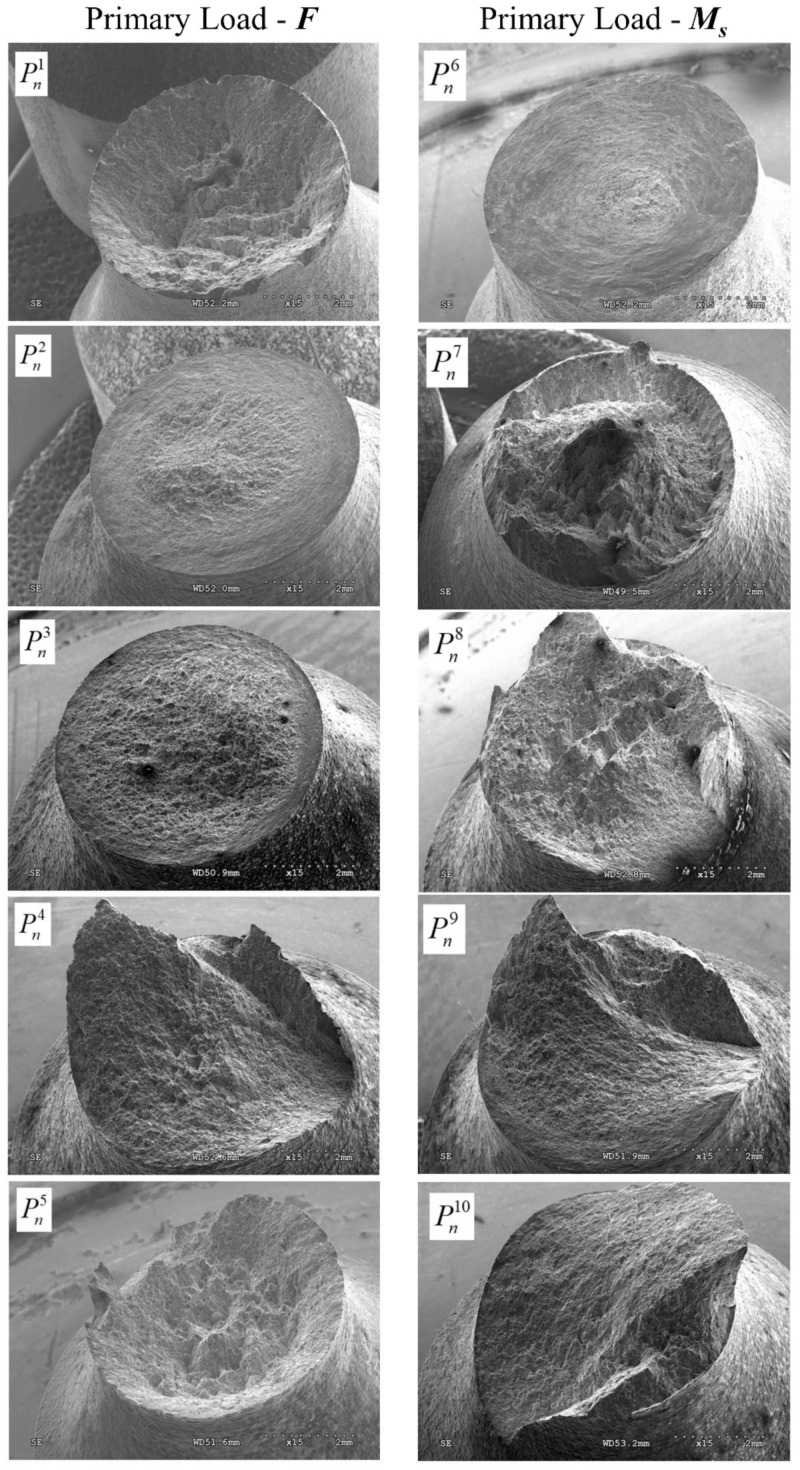
Fracture surface of notched specimens with a notch radius *r*_K_ = 8 mm for different load configurations.

On analyzing the fracture surface on a macroscopic scale, it was noticed that the effect of non-proportional loads had a significant influence on the macroscopic form of the fracture surface. Figure 15 shows the obtained fracture surfaces for specimens depending on the selected load configuration. Attention was paid to changing its shape depending on the load scheme.

**Figure 16:**
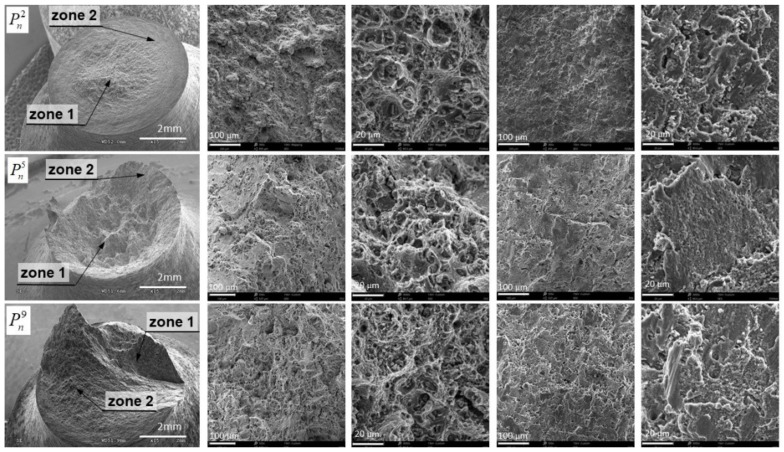
Fracture surfaces for the specimen with a notch radius *r*_K_ = 8 mm.

In the case of load configuration $P_{n}^{2}$, we can clearly distinguish two zones (Figure 16), which confirmed a diversified fracture mechanism. In the central area (Zone 1), dimples were visible after the deformation of pores in the direction of the tensile load, which may indicate the dominance of the maximum principal stresses (or strain) in the fracture process. On the external area of the fracture surface (Zone 2), the dimples were oriented in the direction of rotation, which resulted from the applied load in the form of a torsional moment. It should be assumed that the maximum shear stresses determined the failure in this area. The shape of the dimples was similar to that obtained in the test of pure torsion of a smooth specimen. The share of the central area in the whole fracture surface increased at the expense of the external area for the load case $P_{n}^{3}$. For the load scheme $P_{n}^{4}$, the shape of the fracture surface changed completely, taking the form of a spatial surface on which it is difficult to specify the zones with different fracture mechanisms. A similar shape was also obtained for scheme $P_{n}^{9}$, which showed that with these load configurations, the fracture mechanisms were close together. In the case of load in the configuration $P_{n}^{5}$, the fracture surface was almost identical to that obtained under the load $P_{n}^{1}$, which was characterized by the typical shape obtained from the tension of cylindrical elements made of ductile materials. Therefore, it should be assumed that with such a high level of tensile force $P_{n}^{5}$, the secondary load in the form of torsion had little effect on the shape of the fracture surface and working fracture mechanisms. For load configurations where the primary load was torsional moment, each fracture surface obtained differed from the others. Therefore, it should be assumed that configurations from $P_{n}^{6}$ to $P_{n}^{10}$, where the torsion moment was the primary load, had a key effect on the fracture mechanisms during decohesion of the specimen.

**Figure 17:**
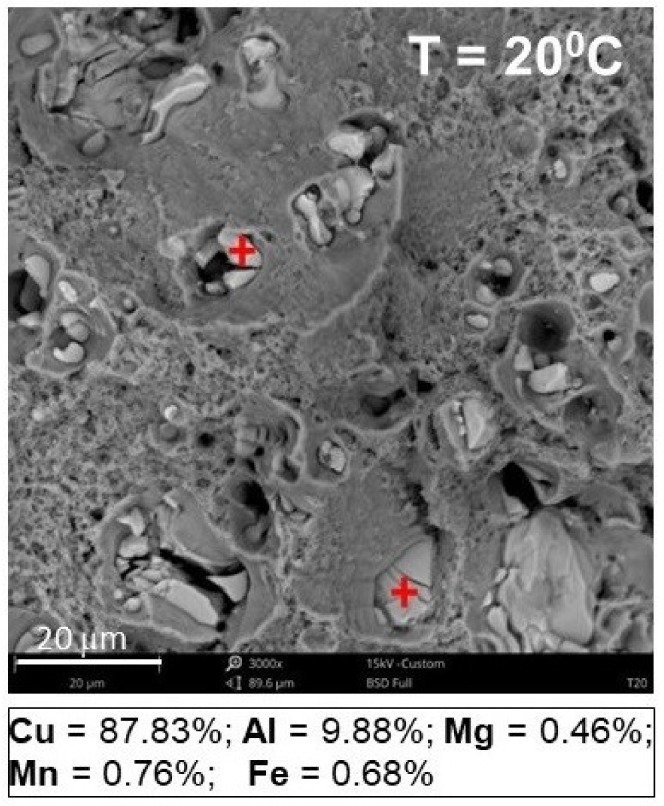
EDS analysis results.

It was observed that on each fracture surface at the bottom of the majority of dimples created, there were numerous precipitates (elements of the second phase) [35]. Based on the analysis of the chemical composition (Figure 17) by means of an integrated EDS detector (energy dispersive spectroscopy), the weight percentage of Cu (copper) at the level of 90% in precipitates of the secondary phase was determined.

During loading, they inhibit the dislocation mobility, which causes a concentration of stresses in their surroundings. When the concentrated stresses reached a critical value, the precipitates separated from the matrix or fractured themselves. As a result, more voids were created. After their coalescence, a new fracture occurred, which caused strong nonlinearity. 

## 4. Conclusions

This paper presents the influence of non-proportional tensile–torsional loads on the fracture process of axisymmetric specimens with a circumferential notch made of aluminum alloy ENAW_2024-T351. Results obtained from the investigation showed that the notch radius and load scheme had a significant influence on the shape of the fracture surface. It was observed that the fracture surface became more irregular and spatial when the notch radius increased, and was characterized by a multi-surface of shapes. In the case when the primary load was tensile force *F*, it was observed that the shapes of some surfaces were similar. For these load configurations from pure torsion to pure tension, it was observed that the shape of the fracture surface changed from planar to a cup-cone shape. On the other hand, when the initial load was the torsional moment, each of the analyzed fracture surfaces was different and was characterized by a spatial, irregular shape in addition to pure torsion. The presented results in dependence on time illustrated the behavior of the secondary load during the primary load. Thanks to this, a relationship describing the change in the critical values of non-proportional load components causing fracture initiation in the material was developed. These results were compared with the results obtained for proportional loads, showing that the configuration of non-proportional loads was more disadvantageous in the load transfer, because they caused fracture initiation in the material faster than in proportional loads. It should also be noted that the increase in the notch radius slightly decreased the critical tensile load and increased the critical torsional moment causing fracture initiation. However, the increase in the notch radius had a large influence on the elongation and torsional angle of the gauge length of the specimen, even 50% in the case of elongation and 65% in the case of the torsion angle. It was presented that for all tested notches, the nonlinear increase in the torsional angle (*φ*) occurred until failure, whereas torsional moment (*M*_s_) remained at a constant level. This was obtained for the configuration where torsional moment was the primary load. The same situation was observed in cases at a constant level of force *F* load, where the elongation of the gauge length *u* in the tested specimen increased linearly. On the basis of the macro and microscopic analysis of the fracture surface, the analyzed material showed two types of fracture mechanisms (for tensile and shear) that caused decohesion.

The obtained results may be the basis for the numerical modeling and formulation of the ductile fracture criterion under the conditions of complex non-proportional loads, or may be used to verify existing criterions.

## Figures and Tables

**Table 1:** Chemical composition of the analyzed alloy.

Component	Si	Fe	Cu	Mn	Mg	Cr	Zn	Ti
**Amount (%)**	0.13	0.25	4.4	0.62	1.7	0.01	0.08	0.05
**Amount (wt.%)**	0.3	0.5	4.9	0.8	1.5	0.1	0.25	0.15

**Table 2:** Values of the basic strength parameters of the ENAW_2024-T351 aluminum alloy and critical values of stress and strain.

*d*_0_ (mm)	*F*_c_ (kN)	*F*_max_ (kN)	*d*_f_ (mm)	*u*_c_ (mm)	*ϕ*_c_ (°)	*M*_c_ = *M*_max_ (N·m)	*E* (GPa)	*R*_e_ (MPa)	*R*_m_ (MPa)	ε (%)	$\varepsilon_{f}$
6	15.92 ± 0.1	16.08 ± 0.09	5.41 ± 0.01	3.46 ± 0.02	58.65 ± 0.02	20.40 ± 0.4	74	424	568	13.8	0.21

**Table 3:** Non-proportional loading cases used in the research.

Loading	Load Cases				
*Tension*	$P_{n}^{1}$	$P_{n}^{2}$	$P_{n}^{3}$	$P_{n}^{4}$	$P_{n}^{5}$
	$F_{C}^{1}=F_{\max}$	$F_{C}^{2}=0.3F_{\max}$	$F_{C}^{3}=0.5F_{\max}$	$F_{C}^{4}=0.7F_{\max}$	$F_{C}^{5}=0.9F_{\max}$
*Torsion*	$P_{n}^{6}$	$P_{n}^{7}$	$P_{n}^{8}$	$P_{n}^{9}$	$P_{n}^{10}$
	$M_{C}^{6}=M_{\max}$	$M_{C}^{7}=0.3M_{\max}$	$M_{C}^{8}=0.5M_{\max}$	$M_{C}^{9}=0.7M_{\max}$	$M_{C}^{10}=0.9M_{\max}$

**Table 4:** Results of fracture for specimens with notches under non-proportional load.

*r*_K_ (mm)	Load	$P_{n}^{1}$	$P_{n}^{2}$	$P_{n}^{3}$	$P_{n}^{4}$	$P_{n}^{5}$	$P_{n}^{6}$	$P_{n}^{7}$	$P_{n}^{8}$	$P_{n}^{9}$	$P_{n}^{10}$
0.5	$F_{c}^{i}$	20.72	6.04	10.06	14.09	18.11	0.00	19.03	17.77	15.24	6.96
	$M_{c}^{i}$	0.00	17.27	15.77	13.30	9.31	18.85	5.71	9.42	13.20	16.95
	$u_{i}^{}$	0.76	0.16	0.32	0.52	0.74	0.12	0.72	0.67	0.53	0.41
	$\varphi_{i}$	0.07	12.31	11.48	10.48	6.98	18.97	5.04	8.88	13.69	16.38
2	$F_{c}^{i}$	19.15	5.75	9.57	13.39	17.20	0.00	18.36	16.71	14.14	7.17
	$M_{c}^{i}$	0.00	18.45	16.96	13.20	8.24	20.54	5.86	9.74	13.59	17.43
	$u_{i}^{}$	1.18	0.19	0.39	0.63	0.91	0.13	0.91	0.81	0.63	0.21
	$\varphi_{i}$	0.16	22.34	20.81	17.68	11.09	23.21	10.72	17.05	20.96	21.94
4	$F_{c}^{i}$	18.55	5.56	8.92	13.01	16.72	0.00	18.77	17.22	14.39	8.07
	$M_{c}^{i}$	0.00	19.20	15.77	14.07	10.11	19.80	5.99	9.92	14.00	17.75
	$u_{i}^{}$	1.27	0.19	0.41	0.59	0.85	0.11	0.89	0.79	0.60	0.51
	$\varphi_{i}$	0.13	22.05	19.38	18.71	14.79	37.29	10.87	19.88	25.16	28.29
8	$F_{c}^{i}$	17.72	5.37	8.93	12.47	16.04	0.00	17.06	15.54	12.06	7.02
	$M_{c}^{i}$	0.00	19.20	15.77	14.07	10.11	20.32	6.10	8.98	14.03	18.03
	$u_{i}^{}$	1.49	0.21	0.55	0.92	1.42	0.01	1.64	0.93	0.85	0.49
	$\varphi_{i}$	0.09	26.71	35.36	21.31	14.41	50.63	19.56	32.52	36.62	42.86
30	$F_{c}^{i}$	17.71	5.31	8.86	12.41	15.94	0.00	16.29	14.26	10.82	4.09
	$M_{c}^{i}$	0.00	18.73	16.08	12.59	6.42	20.67	6.18	10.96	14.33	18.39
	$u_{i}^{}$	1.51	0.38	0.71	1.14	1.33	0.03	2.03	1.77	0.82	0.29
	$\varphi_{i}$	0.08	59.79	45.15	38.72	16.6	54.78	34.05	41.66	63.05	65.12

$F_{c}^{i}$ (kN); $M_{c}^{i}$ (N·m); $u_{i}^{}$ (mm); $\varphi_{i}$ (°).

**Table 5:** Values of exponential parameters used in Equation (2).

Load Case	*A*	*B*
Proportional	2	4
Non-proportional	1.65	2

## Nomenclature

*d*_f_	measurable diameter after the test (mm)
*E*	Young Modulus (GPa)
*F*	tension load (N)
*F*_c_	critical tension load (N)
*L*_0_	length of gauge length of smooth specimen (mm)
*M*	torsional moment (N·m)
*M*_c_	critical torsional moment (N·m)
*R*_e_	yield strength (MPa)
*r*_K_	notch radius (mm)
*R*_m_	ultimate tensile strength (MPa)
*u*_c_	critical elongation of gauge length of specimen (mm)
Δ*u*	elongation of gauge length of specimen (mm)
*ε*	percentage elongation after fracture (%)
*ε*_f_	critical failure strain
*φ*	torsional angle of gauge length of specimen (°)
*φ*_c_	critical torsional angle of gauge length of specimen (°)
*ϕ*_d_	dimension of gauge length of smooth specimen (mm)
*ϕ*_K_	dimension of specimen in notch root (mm)

## References

[1] Gandt A.F. (2004). Fundamentals of Structural Integrity—Damage Tolerant Design and Nondestructive Evaluation.

[2] Lukács J. Application of Conditional Fracture Toughness Values for Reliability Assessment Calculations. Proceedings of the Design, Fabrication and Economy of Metal Structures.

[3] BS 7910 (1999). Guide to Methods for Assessing the Acceptability of Flaws in Metallic Structures.

[4] Algarni M., Bai Y., Choi Y. (2015). A study of Inconel 718 dependency on stress triaxiality and Lode angle in plastic deformation and ductile fracture. Eng. Fract. Mech..

[5] Bai Y., Teng X., Wierzbicki T. (2009). On the Application of Stress Triaxiality Formula for Plane Strain Fracture Testing. J. Eng. Mater. Technol..

[6] Bao Y., Treitler R. (2004). Ductile crack formation on notched Al2024-T351 bars under compression–tension loading. Mater. Sci. Eng. A.

[7] Bao Y., Wierzbicki T. (2004). On fracture locus in the equivalent strain and stress triaxiality space. Int. J. Mech. Sci..

[8] Brünig M., Gerke S., Hagenbrock V. (2013). Micro-mechanical studies on the effect of the stress triaxiality and the Lode parameter on ductile damage. Int. J. Plast..

[9] Gao X., Zhang T., Zhou J., Graham S.M., Hayden M., Roe C. (2011). On stress-state dependent plasticity modeling: Significance of the hydrostatic stress, the third invariant of stress deviator and the non-associated flow rule. Int. J. Plast..

[10] Papasidero J., Doquet V., Mohr D. (2015). Ductile fracture of aluminum 2024-T351 under proportional and non-proportional multi-axial loading: Bao–Wierzbicki results revisited. Int. J. Solids Struct..

[11] Derpeński Ł., Seweryn A. (2011). Experimental research into fracture of EN-AW 2024 and EN-AW 2007 aluminium alloy specimens with notches subjected to tension. Exp. Mech..

[12] Derpeński Ł., Seweryn A. (2016). Ductile fracture of EN-AW 2024 aluminum alloy specimens with notches under biaxial loading. Part 1—Experimental research. Theor. Appl. Fract. Mech..

[13] Wierzbicki T., Bao Y., Lee Y.-W., Bai Y. (2005). Calibration and evaluation of seven fracture models. Int. J. Mech. Sci..

[14] Papasidero J., Doquet V., Lepeer S. (2014). Multiscale investigation of ductile fracture mechanisms and strain localization under shear loading in 2024-T351 aluminum alloy and 36NiCrMo16 steel. Mater. Sci. Eng. A.

[15] Faleskog J., Barsoum I. (2013). Tension–torsion fracture experiments—Part I: Experiments and a procedure to evaluate the equivalent plastic strain. Int. J. Solids Struct..

[16] Papasidero J., Doquet V., Mohr D. (2014). Determination of the Effect of Stress State on the Onset of Ductile Fracture through Tension-Torsion Experiments. Exp. Mech..

[17] Cortese L., Nalli F., Rossi M. (2016). A nonlinear model for ductile damage accumulation under multiaxial non-proportional loading conditions. Int. J. Plast..

[18] Algarni M., Choi Y., Bai Y. (2017). A unified material model for multiaxial ductile fracture and extremely low cycle fatigue of Inconel 718. Int. J. Fatigue.

[19] Gerke S., Zistl M., Bhardwaj A., Brünig M. (2019). Experiments with the X0-specimen on the effect of non-proportional loading paths on damage and fracture mechanisms in aluminum alloys. Int. J. Solids Struct..

[20] Zistl M., Gerke S., Brunig M. (2018). Biaxial experiments on the effect of non-proportional loading paths on damage and fracture behaviour of ductile metals. Proc. Struct. Integr..

[21] Haltom S., Kyriakides S., Ravi-Chandar K. (2013). Ductile failure under combined shear and tension. Int. J. Solids Struct..

[22] Beese A.M., Luo M., Li Y., Bai Y., Wierzbicki T. (2010). Partially coupled anisotropic fracture model for aluminum sheets. Eng. Fract. Mech..

[23] Roth C.C., Mohr D. (2014). Effect of strain rate on ductile fracture initiation in advanced high strength steel sheets: Experiments and modeling. Int. J. Plast..

[24] Toribio J., Vergara D., Lorenzo M. (2016). Influence of Loading Rate on the Hydrogen-Assisted Micro-Damage in Bluntly Notched Samples of Pearlitic Steel. Metals.

[25] Smith E. (2004). The elastic stress distribution near a circular cylindrical notch due to external dislocations. Int. J. Eng. Sci..

[26] Sanyal G., Das A., Singh J., Chakravartty J. (2015). Effect of notch geometry on fracture features. Mater. Sci. Eng. A.

[27] Peron M., Razavi S.M.J., Torgersen J., Berto F. (2017). Fracture Assessment of PEEK under Static Loading by Means of the Local Strain Energy Density. Materials.

[28] Shokuhfar A., Nejadseyfi O. (2014). A comparison of the effects of severe plastic deformation and heat treatment on the tensile properties and impact toughness of aluminum alloy 6061. Mater. Sci. Eng. A.

[29] Mostafavi M., Smith D.J., Pavier M.J. (2011). Fracture of aluminium alloy 2024 under biaxial and triaxial loading. Eng. Fract. Mech..

[30] Khan A.S., Liu H. (2012). A new approach for ductile fracture prediction on Al 2024-T351 alloy. Int. J. Plast..

[31] Wagoner R.H., Laukonis J.V. (1983). Plastic behavior of aluminum-killed steel following plane-strain deformation. Met. Mater. Trans. B.

[32] Hosford W.F., Caddell R.M. (2014). Metal Forming: Mechanics and Metallurgy.

[33] Wang H., Yan Y., Han F., Wan M. (2017). Experimental and theoretical investigations of the forming limit of 5754O aluminum alloy sheet under different combined loading paths. Int. J. Mech. Sci..

[34] Brindgman P.W. (1964). Studies in Large Plastic Flow and Fracture with Special Emphasis on the Effect of Hydrostatic Pressure.

[35] Derpenski Ł., Seweryn A. (2019). Ductile fracture of notched aluminum alloy specimens under elevated temperature. Part 1—Experimental research. Theor. Appl. Fract. Mech..

